# ED50 value of remifentanil in inhibiting coughing during extubation in children with snoring

**DOI:** 10.3389/fphar.2022.965354

**Published:** 2022-09-07

**Authors:** Dong-Mei Chen, Min Yang, Xiao-Ye Ren, Shi-Peng Su, Ling Li, Qi Jia, Hai-Yan Zhong, Jian-Ping Yan

**Affiliations:** ^1^ Department of Anesthesiology, Affiliated Hospital of Inner Mongolia Medical University, Hohhot, China; ^2^ Department of Anesthesiology, Peking University Cancer Hospital & Institute, Beijing, China; ^3^ Department of General Surgery, Huhhot First Hospital, Hohhot, China

**Keywords:** remifentanil, extubation, children, low-dose infusion, cough

## Abstract

**Objective:** This study aimed to determine the effective dose 50% (ED50) value of remifentanil in inhibiting coughing during extubation in children with snoring.

**Methods:** The subjects were children who scored a grade I in the American Society of Anesthesiology (ASA) metric and who were undergoing tonsillectomy (with or without adenoidectomy) under general anesthesia. Using Dixon’s up-and-down sequential method, the initial infusion rate of remifentanil was 0.06 μg/kg/min, and the difference between the infusion rates of the two adjacent groups was 0.01 μg/kg/min. If a child had no cough response during extubation, the infusion rate for the next child was reduced by 0.01 μg/kg/min. If that child had cough response, the infusion rate for the next child was increased by 0.01 μg/kg/min, and the test was terminated when seven pairs of children with positive-negative alternating results were obtained. The ED50 value and its 95% confidence interval (CI) were calculated by probit regression. The times for extubation, awakening, agitation, and respiratory complications after extubation were compared between the two groups.

**Results:** 1) The ED50 value of a continuous infusion of remifentanil required to inhibit the cough response of children during extubation was 0.042 μg/kg/min, and the 95% confidence interval was 0.025–0.062 μg/kg/min. 2) The total dosage and infusion rate of remifentanil in the cough suppression group were higher than those in the cough group (*p* < 0.05), but the differences in the times for extubating and awakening between the two groups were not statistically significant (*p* > 0.05). 3) There was no correlation between the infusion rate of remifentanil and the time for extubating and awakening in the cough suppression group; the r values were 0.13 and 0.12, respectively, and *p* > 0.05. 4) The differences in postoperative respiratory complications between the two groups were not statistically significant (*p* > 0.05).

**Conclusion:** The ED50 value of a continuous infusion of remifentanil required to inhibit the cough response of children during extubation after tonsillectomy (with or without adenoidectomy) was 0.042 μg/kg/min, and a low-dose infusion of remifentanil does not affect the times for awakening and extubating in children.

## 1 Introduction

Coughing that occurs during extubation following general anesthesia is a very common clinical phenomenon, with an incidence of 50%–94.7% (McHardy and Chung, 1999). Although the duration of coughing is brief and recovery of the coughing reflex can prevent aspiration, it can also cause hemodynamic fluctuations, arrhythmias, hypoxemia, laryngeal spasm, and bronchospasm (Yang et al., 2017). Especially after tonsillectomy (with or without adenoidectomy) due to snoring in children, the throat is more sensitive due to surgical stimulation. Continuous coughing increases children’s airway reactivity and causes them to be more prone to respiratory complications such as laryngeal spasm, hypoxemia, and atelectasis, which can result in serious consequences (Stone, 2000). Avoiding the occurrence of the cough response and enabling children to pass the extubation period safely was our goal.

The purpose of this study was to determine the effective dose 50% (ED50) and 95% confidence interval (CI) of remifentanil needed to inhibit the cough response during extubation after tonsillectomy (with or without adenoidectomy) under total intravenous anesthesia and to provide a reference for clinical work to enrich the strategy of safe extubation.

### 1.1 Information and methods

#### 1.1.1 General information

This study was approved by the ethics committee of our hospital, and all parents or guardians of the patients provided a signed informed consent form. Children aged 3–8 years with a grade I American Society of Anesthesiology (ASA) score who underwent tonsillectomy (with or without adenoidectomy) were enrolled in this study. Exclusion criteria: Patients with an upper respiratory tract infection within 2 weeks of surgery; patients with a history of an opioid allergy; patients with asthma, allergy, and other hypersensitivity reactions; patients with accidental bleeding during surgery; patients who required more than two intubation attempts.

After the patients entered the operating room, electrocardiogram, arterial oxygen saturation (SpO_2_), noninvasive blood pressure, and bispectral index (BIS) were routinely monitored. Venous access was initiated. Endotracheal intubation was performed after intravenous injections of propofol (2–3 mg/kg), remifentanil hydrochloride (2 μg/kg), and rocuronium (0.6 mg/kg) were administered. Patients are given remifentanil and propofol at actual weight, rocuronium are given at standard weight. We all use visual laryngoscope for intubation. The respiratory parameters were adjusted according to P_ET_CO_2_ to maintain P_ET_CO_2_ between 35 and 45 mmHg. Propofol (6–8 mg/kg/h) and remifentanil (0.2–0.3 μg/kg/min) were used for intraoperative maintenance, muscle relaxants were not added for the duration of the surgery, and the BIS value was controlled within 40–60. Ketorolac (0.5 mg/kg) was given intravenously 5 min before the end of the operation, and the maximum dose was no more than 15 mg. At the end of the operation, the surgeon performed local anesthesia with lidocaine in the tonsil recess to achieve postoperative analgesia, and we adopted multi-mode analgesia. When the self-retaining laryngoscope was removed, the infusion of propofol was stopped, and the infusion rate of remifentanil was adjusted to the specified value until extubation. While the patient was still under deep anesthesia, oral sputum suction was gently performed. After sputum suction, the ventilator was turned off to stimulate breathing. If the child does not breathe autonomously for a long time, we will carry out manual assisted breathing. After the recovery of spontaneous breathing, neostigmine (0.02–0.04 mg/kg) and atropine (0.01–0.02 mg/kg) were given routinely for antagonism. When the patient’s tidal volume reached 6 ml/kg, their respiratory rate was ≥11 times/min, their blood oxygen saturation was maintained at more than 95% under deoxygenation for more than 3 min, and their P_ET_CO_2_ value was less than 50 mmHg, deflation was performed with a cuff, and the endotracheal tube was pulled out. The same extubation standard was used for all children.

## 2 Methods

The Dixon up-down sequential method was used to set the dose of remifentanil. According to the research of Park et al. (Park et al., 2015), the ED95 value of remifentanil in inhibiting cough response during extubation in children is 0.06 μg/kg/min. Therefore, the dose of remifentanil for the first child in this study was set at 0.06 μg/kg/min and the infusion remained at this rate for the whole recovery period, until the endotracheal tube was removed. If the child had no cough response during extubation, the infusion rate for the next child was reduced by 0.01 μg/kg/min. If that child had cough response during extubation, the infusion rate for the next child was increased by 0.01 μg/kg/min. The test was terminated when seven pairs of children with positive-negative alternating results were obtained. In order to make the experimental results more authentic and reliable, the sample size was appropriately increased.

### 2.1 Observation indexes

Multiple observations were recorded, including: the operation time, the total infusion dose of remifentanil, the infusion rate of remifentanil, the extubation time, the awakening time, the BIS value at extubation, the cough response, the agitation score, the pain score, the time the patient left the operating room, and the number of cases with an SpO_2_< 95% after returning to the ward. The agitation score was divided into five grades: one point: quiet sleep; two points: sober and calm; three points: irascible, irritable, and crying; four points: uncomfortable and uncontrollable crying; five points: unable to be quiet, confused, and delirious. The pain score was calculated using the Face, Legs, Activity, Cry, Consolability (FLACC) scoring standard: 0 point indicated no pain; 1–3 points indicated slight discomfort; 4–6 points indicated moderate pain; 7–9 points indicated severe pain, and 10 points indicated unbearable pain.

Evaluation criteria of recovery period indicators: The time for extubation was measured from the time that propofol was stopped to when the endotracheal tube was removed. The time for awakening was measured from the time that propofol was stopped until the child opened his/her eyes or had voluntary limb activities, and the time for leaving the operation room was measured by the time the child opened his/her eyes to when they left the operating room.

### 2.2 Statistical analysis

Data were statistically analyzed using the statistical software package SPSS 25.0. Normally distributed measurement data were expressed as mean ± standard deviation ( × ± SD) and compared between the two groups using a *t*-test. Non-normally distributed measurement data were expressed as the median and compared between groups using a non-parametric test. The balance of gender between the two groups was evaluated using an X^2^ test. *p* < 0.05 was considered statistically significant. A probit regression model was used to calculate the ED50 value and 95% CI of inhibit cough response during extubation.

## 3 Results

### 3.1 ED50 value of remifentanil in inhibiting cough response during extubation

**FIGURE 1:**
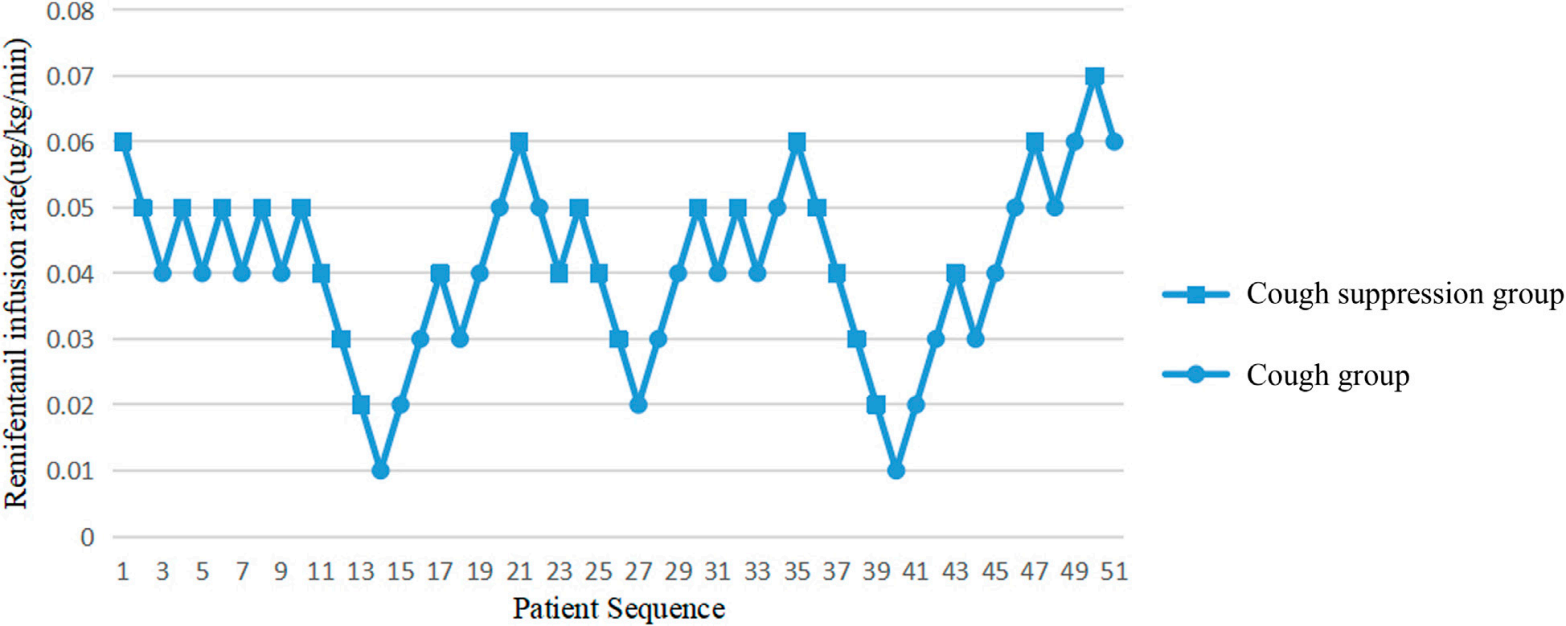
Infusion rate of remifentanil and extubation response in children.

A probit model calculated that the ED50 value of the continuous infusion of remifentanil required to inhibit the cough response of children during extubation was 0.042 μg/kg/min, and the 95% CI was 0.025–0.062 μg/kg/min. The extubation responses of children and the corresponding infusion rates of remifentanil are shown in Figure 1.

### 3.2 Differences of children in the cough group and the cough suppression group

There were no significant differences in gender, age, weight, and operation time between the cough group and the cough suppression group (*p* > 0.05, Table 1).

**TABLE 1:** Comparison of general situations between two groups of children.

Group	Gender (male/female)	Age (years old)	Weight (kg)	Operation time (min)
Cough group (*n* = 26)	14/12	4.5 (4.8)	19.5 (16,29.75)	35 (26.75,42.5)
Cough suppression group (*n* = 25)	15/10	4 (4.6.5)	19 (16,25.5)	27 (23.5,40)

### 3.3 Intraoperative and recovery indexes

The total dosage of remifentanil in the cough suppression group was higher than that of the cough group (*p* < 0.05), and the difference was statistically significant. The infusion rate of remifentanil was 0.037 ± 0.013 μg/kg/min in the cough group, and 0.046 ± 0.013 μg/kg/min in the cough suppression group, which was higher in the cough suppression group than in the cough group (*p* < 0.05), and the difference was statistically significant (*p* < 0.05). The time for extubation and the time for awakening were longer in the cough suppression group than in the cough group (*p* > 0.05), and the differences were not statistically significant. The differences in BIS values between the two groups during extubation and the time the operating room was left were not statistically significant between the two groups (*p* > 0.05, Table 2).

**TABLE 2:** Comparison of intraoperative and recovery indexes.

Group	Total dosage of remifentanil (mg)	Remifentanil infusion rate (ug/kg/min)	Time for extubation (min)	Time for awakening (min)	BIS value during extubation	Time of leaving operation room and returning to ward (min)
Cough group (*n* = 26)	0.17 (0.14.0.20)	0.037 ± 0.013	12.42 ± 5.27	13.23 ± 5.86	73.92 ± 2.56	4 (3, 5.25)
Cough suppression group (*n* = 25)	0.20^a^ (0.16.0.32)	0.046 ± 0.013^b^	15.24 ± 4.88	15.80 ± 5.08	74.52 ± 2.37	4 (2, 4.5)

a Notes: *P* < 0.05 compared with cough group.

b *P*<0.05 compared with cough group.

### 3.4 Correlation between the remifentanil infusion rate, the time for awakening, and the time for extubation in the cough suppression group after surgery

**FIGURE 2:**
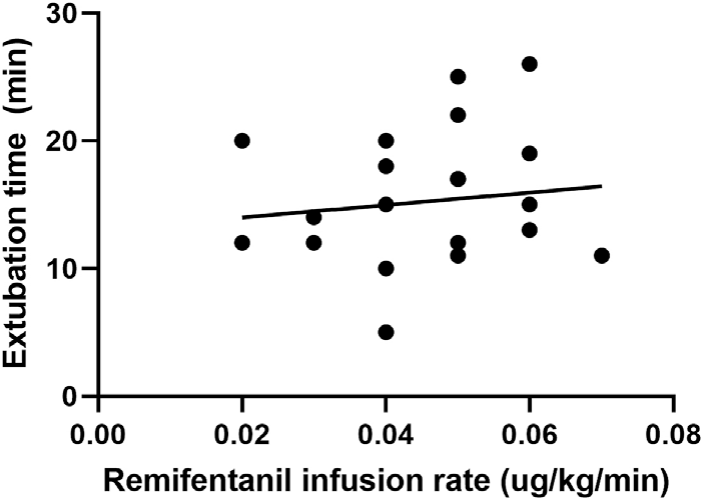
Scatter plot of remifentanil infusion rate and the time for extubation in the cough suppression group.

**FIGURE 3:**
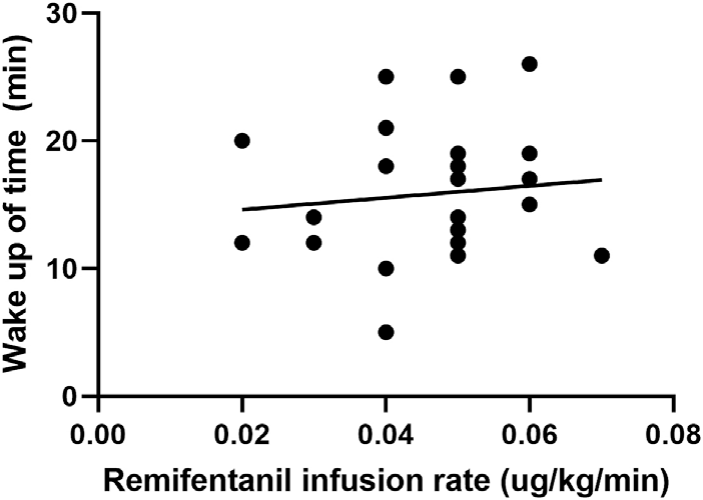
Scatter plot of remifentanil infusion rate and the time for awakening in the cough suppression group.

In the cough suppression group, the scatter plots of infusion rates of remifentanil, the time for extubation, and the time for awakening of the children are shown in Figures 2, 3, respectively, and the correlation coefficient is shown in Table 3. There was no correlation for all (*p* > 0.05).

**TABLE 3:** Correlation of the infusion rate of remifentanil with the time for extubation and the time for awakening in the cough suppression group.

Index	Remifentanil infusion rate in the cough suppression group	
	r	*p*
Time for extubation	0.13	0.55
Time for awakening	0.12	0.58

### 3.5 Postoperative complications

There were no significant differences in the postoperative agitation scores, pain scores, or persistent cough after extubation between the two groups (*p* > 0.05). None of the children had nausea or vomiting, delayed awakening, laryngeal spasm, or postoperative reflux disease, and SpO_2_< 95% (Table 4).

**TABLE 4:** Comparison of postoperative complications between the two groups.

Group	Agitation score	Pain score	Nausea and vomiting	Delayed awakening	Laryngeal spasm	Persistent cough after extubation	SpO_2_ <95% in the ward after operation
Cough group (*n* = 26)	2 (2,2)	0 (0,0)	0	0	0	2	0
Cough suppression group (*n* = 25)	2 (2,2)	0 (0,1)	0	0	0	1	0

## 4 Discussion

Most adult studies employ a target-controlled infusion (TCI) method with the infusion of remifentanil to inhibit coughing. However, the Minto model used in TCI is not suitable for patients younger than 20 years old, as the infusion dose in children can only be adjusted according to their weight (Minto et al., 1997). In this study, the infusion mode of remifentanil was a continuous intravenous infusion.

In this study, it was measured that the ED50 value of the continuous infusion of remifentanil required to inhibit the cough response of snoring children who underwent tonsillectomy (with or without adenoidectomy) under general anesthesia during extubation was 0.042 μg/kg/min, and the 95% CI was 0.025–0.062 μg/kg/min. There were no adverse reactions such as hypoxemia, laryngeal spasm, or delayed recovery in children, so it was a safe and effective extubation strategy.

The infusion rates and total dosages of remifentanil were higher in the cough suppression group than in the cough group. The results suggest that a relatively high dose of remifentanil was used in the cough suppression group, and there was a significant difference in extubation response. This suggests that remifentanil can effectively inhibit the cough response, which is consistent with the results of a previous study (Tschiedel et al., 2021). Although the times for extubation and the time for awakening were slightly longer in the cough suppression group than in the cough group, the differences between the two groups were not statistically significant. Further analysis was made for the correlation of the remifentanil infusion rate between the time for extubation and the time for awakening in children in the cough suppression group, and the results revealed that the correlation coefficient between the remifentanil infusion rate and the time for extubation was *r* = 0.13 (*p* > 0.05). The correlation coefficient between the remifentanil infusion rate and the time for awakening was *r* = 0.12 (*p* > 0.05). The results suggest that there is no correlation of a low-dose infusion of remifentanil with the time for extubation and the time for awakening. In the cough suppression group, the infusion rate of remifentanil did not delay the time for extubation and the time for awakening. Yuguang Huang et al. (Wang et al., 2014) studied that the TCI of a low-dose remifentanil infusion during extubation could improve the quality of extubation during awakening. They also found that a remifentanil infusion did not affect the extubation time and the time for awakening, and this was consistent with the results of the present study. Kwon et al. (2019) revealed in their study that a remifentanil infusion did not affect the time for awakening. However, it is necessary to pay attention to the adverse reactions of opioids during high-dose infusion (Zhang et al., 2017), as low doses of remifentanil could reduce the incidence of related complications (Bagshaw et al., 2020).

Previous studies revealed that when the BIS value was 70–75 during extubation, the children’s heart rates and blood pressures fluctuated slightly and they had less agitation, which is the appropriate anesthesia depth for extubation (Gao et al., 2015). At this depth of anesthesia, the swallowing and cough reflexes recovered well, and the children were fully awake 3–5 min after extubation. It is safest to extubate at this depth of anesthesia (Xin and Zhang, 2012). In this study, the BIS value during the recovery period was also monitored. The results revealed that the BIS values of the cough suppression and cough groups during extubation were between 71 and 76. When extubation was carried out at this depth of anesthesia, the children had less agitation and the time from extubation to complete wakefulness was short. After about 4 min, the children woke up, left the operating room and were returned to the ward. It is further verified that a BIS value between 71 and 76 is a relatively safe and appropriate level for extubation. There were no significant differences in BIS values between the two groups, suggesting that in the cough suppression group, a relatively higher infusion rate of remifentanil can effectively inhibit coughing, avoiding the impact of different sedation depths on coughing occurrences within the two groups.

In this study, in order to eliminate the different stress responses caused by different types of surgery, only one type of surgery was selected, and the sample size of the study was insufficient. Future research is needed to expand the number of cases and select different types of surgery to improve the accuracy and scope of the application of the research results.

In summary, the ED50 value of a continuous infusion of remifentanil to inhibit the cough response of children who underwent tonsillectomy (with or without adenoidectomy) to treat snoring during extubation was 0.042 μg/kg/min; the 95% CI was 0.025–0.062 μg/kg/min, and a low-dose infusion of remifentanil does not affect the time for awakening and the time for extubation in children.

## Data Availability

The original contributions presented in the study are included in the article/Supplementary Material, further inquiries can be directed to the corresponding authors.
